# Physiological Remediation of Cobalt Ferrite Nanoparticles by Ferritin

**DOI:** 10.1038/srep40075

**Published:** 2017-01-09

**Authors:** Jeanne Volatron, Jelena Kolosnjaj-Tabi, Yasir Javed, Quoc Lam Vuong, Yves Gossuin, Sophie Neveu, Nathalie Luciani, Miryana Hémadi, Florent Carn, Damien Alloyeau, Florence Gazeau

**Affiliations:** 1 Laboratoire Matière et Systèmes Complexes, UMR 7057 CNRS/Université Paris Diderot, Sorbonne Paris Cité, 10 rue Alice Domon et Léonie Duquet, 75205 Paris Cedex 13, France; 2 Inserm U970, Paris Cardiovascular Research Center-PARCC/Université Paris-Descartes, Sorbonne Paris Cité, 56 rue Leblanc, 75015 France; 3 Laboratoire Matériaux et Phénomènes Quantiques, UMR 7162 CNRS/Université Paris Diderot, Sorbonne Paris Cité, 10 rue Alice Domon et Léonie Duquet, 75205 Paris Cedex 13, France; 4 Department of Physics, University of Agriculture Faisalabad, Pakistan; 5 Service de Physique Biomédicale, Université de Mons, 20 Place du Parc, 7000 Mons, Belgium; 6 Laboratoire PHENIX, UMR 7195, CNRS/Université Pierre et Marie Curie/ESPCI, 4 place Jussieu, 75252 Paris Cedex 05, France; 7 ITODYS, Interfaces, Traitements, Organisation et Dynamique des Systèmes, UMR 7086 CNRS/Université Paris Diderot, Sorbonne Paris Cité, 15 rue Jean-Antoine de Baïf, 75205 Paris Cedex 13, France

## Abstract

Metallic nanoparticles have been increasingly suggested as prospective therapeutic nanoplatforms, yet their long-term fate and cellular processing in the body is poorly understood. Here we examined the role of an endogenous iron storage protein – namely the ferritin – in the remediation of biodegradable cobalt ferrite magnetic nanoparticles. Structural and elemental analysis of ferritins close to exogenous nanoparticles within spleens and livers of mice injected *in vivo* with cobalt ferrite nanoparticles, suggests the intracellular transfer of degradation-derived cobalt and iron, entrapped within endogenous protein cages. In addition, the capacity of ferritin cages to accommodate and store the degradation products of cobalt ferrite nanoparticles was investigated *in vitro* in the acidic environment mimicking the physiological conditions that are present within the lysosomes. The magnetic, colloidal and structural follow-up of nanoparticles and proteins in the lysosome-like medium confirmed the efficient remediation of nanoparticle-released cobalt and iron ions by ferritins in solution. Metal transfer into ferritins could represent a quintessential process in which biomolecules and homeostasis regulate the local degradation of nanoparticles and recycle their by-products.

The lifecycle of inorganic nanoparticles (NPs) in the body and potentially associated health risks still raise serious concerns. The evaluation of the fate of NPs is important to understand the behavior of particles that have been intentionally injected for medical purposes, but might also help predicting the outcomes related to particles environmental and occupational exposure. NPs made of metallic compounds such as quantum dots, silver, gold, iron and cobalt have been proposed for their potential in imaging and therapy, as well as their widespread technological applications. The exposure to NPs indeed raises questions on their interactions with proteins, intracellular processing, persistence and residence time within the organs^1^,^2^. What kind of mechanisms could be employed by the cell to process NPs? When particles degrade, could the body eliminate or recycle NPs degradation products? Do we have some innate mechanisms that could protect us from potential deleterious effects generated by NPs or their degradation products? Are there some pathways of NP remediation ensured by endogenous proteins?

Iron oxide nanoparticles (IONPs) found their way into the clinics because they are biodegradable and their products of degradation can be processed by the physiological iron metabolism. Studies evaluating the fate of IONPs *in vivo* demonstrated that IONPs lose their magnetic properties over time while being degraded in the acidic environment of lysosomes, mostly in splenic and hepatic macrophages^3^,^4^,^5^. Studies generally converge to a cellular processing of IONPs, which involves the physiological homeostasis pathways for iron^6^,^7^,^8^,^9^. The remediation of iron coming from radiolabelled IONPs can be monitored and quantified by measuring the amount of ^59^Fe incorporated into the hemoglobin of newly formed erythrocytes, which demonstrates that the degradation products of IONPs can join the iron pool of the organism. Intracellular storage of iron is also mediated by ferritin, a protein cage-like structure composed of 24 self-assembled subunits in which up to 4500 iron atoms can be stored^10^,^11^. Moreover, the gene expression of ferritin and other proteins involved in iron homeostasis is upregulated after cell internalization of IONPs^10^,^12^,^13^. Magnetic studies in organs *ex vivo* showed the transformation of the original superparamagnetic properties of IONPs into the magnetic signature of iron-filled ferritins^3^,^11^. At the nanoscale, transmission electron microscopy (TEM) revealed the ubiquitous presence of ferritins close to intracellular IONPs, in spleen, liver and inflammatory macrophages. Therefore ferritin appears involved in the intracellular degradation mechanism of IONPs *in vivo* and in the recycling and storage of iron ions released by NPs^3^,^14^,^15^. Such a scenario could consequently warrant minimal toxicity of iron-based NPs, since the potentially harmful labile iron species generated by the intracellular degradation of IONPs could be captured in its non-toxic form within ferritin proteins. Here we questioned if other metals, such as cobalt coming from the degradation of nanoparticles, could follow the same pathway of physiological remediation by endogenous ferritin.

In order to modulate the magnetic properties of IONPs, partial replacement of ferrous ions with other divalent ions has been suggested, the most prominent example being the introduction of cobalt ions. Cobalt ferrite NPs (CoIONPs) arise interest for theranostic applications, data storage, catalysis, environmental and biodevices as their magnetocristalline anisotropy can be finely tuned by cobalt substitution. This approach is particularly useful for magnetic hyperthermia, which benefits from optimized hyperthermic efficacy at tunable excitation with different magnetic field frequencies^16^,^17^,^18^,^19^,^20^. However little is known about the *in vivo* fate of nanoparticles metallic dopants such as cobalt. The potential toxicity of CoIONPs is a subject of debate with regards to environmental, health and safety issues connected to their application in nanomedicine^21^,^22^,^23^. Natural organic matter as well as cellular media can promote degradation of CoIONPs resulting in possible release of Co^2+^ and Fe^3+^/Fe^2+^ into the environment^24^,^25^. Reports on Co toxicity suggested that Co^2+^ ions are the primary toxic form of Co^26^,^27^. Particularly, some interference with Ca^2+^ entry and signaling has been reported. Therefore the important issues of the dissolution of CoIONPs and of the fate of released ions need to be investigated *in vitro* and *in vivo*.

In this paper we present an ultrastructural follow up of CoIONPs nanoparticles over four months after intravenous injection in mice. Our focus was to get insight into the role of ferritin proteins in the intracellular recycling and remediation of metal oxide nanoparticles and to examine if Co^2+^ and Fe ions released by NPs can be stored into endogenous ferritin proteins. Ferritin proteins are implicated in various metabolic processes^28^, owing to their ability to detoxify, carry, store and release iron in a controlled fashion^29^. It has been shown that ferritins also act as a multifunctional detoxicant for soluble metal ions other than iron^30^,^31^,^32^,^33^. Studies published in the eighties evidenced ferritin’s *in vivo* binding potential for metals such as Cu^2+^, Cd^2+^, Al^2+^, Zn^2+^ and Be^2+^ after administration of their respective salts^32^,^33^,^34^. *In vitro*, the protein shell called apoferritin (ApoF) and the iron-filled protein called holoferritin (HoloF) have different affinities for divalent metal atoms also depending of the pH^31^. Moreover, ApoF was successfully used as a monodisperse and spherical-shaped biotemplate for the mineralization of various inorganic NPs including iron, cobalt, manganese, platinum, zinc, rare earths-doped yttrium-vanadate or gadolinium^19^,^31^,^35^,^36^,^37^,^38^,^39^,^40^,^41^. The *in vitro* versatility of ferritin proteins to encapsulate different metals suggests that they could also play a role *in vivo* in the metabolism of various metallic NPs. While ferritin is considered as a physiological constituent in several tissues, other proteins, such as the metallothionein, are synthesized under metabolic stress in response to highly toxic levels of metal ions, and could also be involved in metallic NPs metabolism^32^. Ferritin and metallothionein both act as chelators-donors of metal ions, but since ferritin is present at a higher constitutive level, it could act as a first line defense against metal ions^32^. While previous studies demonstrated *in vitro* divalent metal binding to ferritins at pH 7.4, the acidic pH of 5.5 nearly abolished the binding of metals other than Fe^2+^ and Cu^2+^ 31. Nevertheless, here we question the possibility of ferritin storing of cobalt, released by CoIONPs *in vitro* and *in vivo*, at a pH of 4.7, which is physiologically present in lysosomes, where NPs are confined and degraded. The mechanism of metal transfer from NPs to endogenous proteins such as ferritin would exemplify a quintessential process in which biomolecules and homeostasis regulate the local degradation of NPs and recycle their by-products.

## Results and Discussion

**Figure 1:**
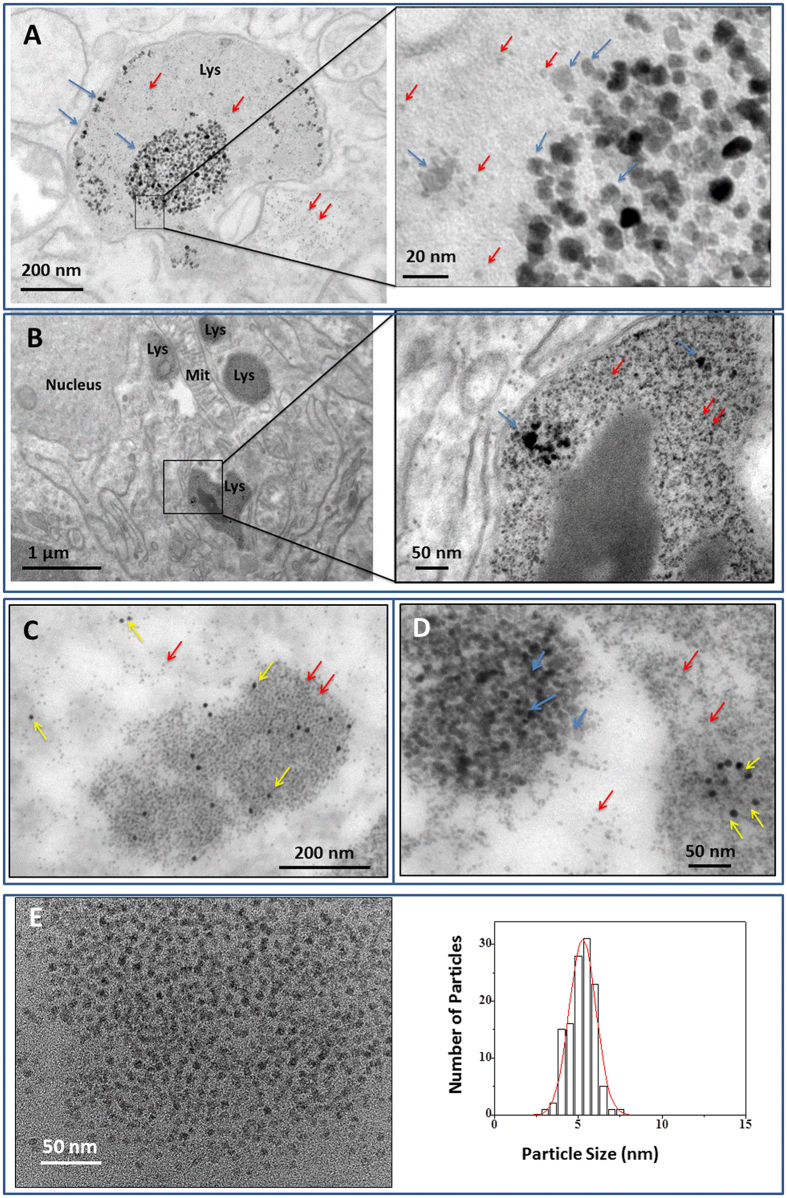
TEM micrographs of spleen samples at day 1 (**A**), day 7 (**B**) and day 42 (**C**,**D**) after intravenous injection of CoIONPs in mice. CoIONPs (indicated with blue arrows) are found in lysosomes (Lys) of macrophages in proximity to iron-filled ferritin proteins (HoloF) (red arrows). In C&D, ferritin proteins are labeled with 10 nm colloidal gold anti-ferritin antibody (yellow arrows). Note the organization of HoloF as large clusters. (**E**) HoloF aggregates in spleen and corresponding size distribution.

**Figure 2:**
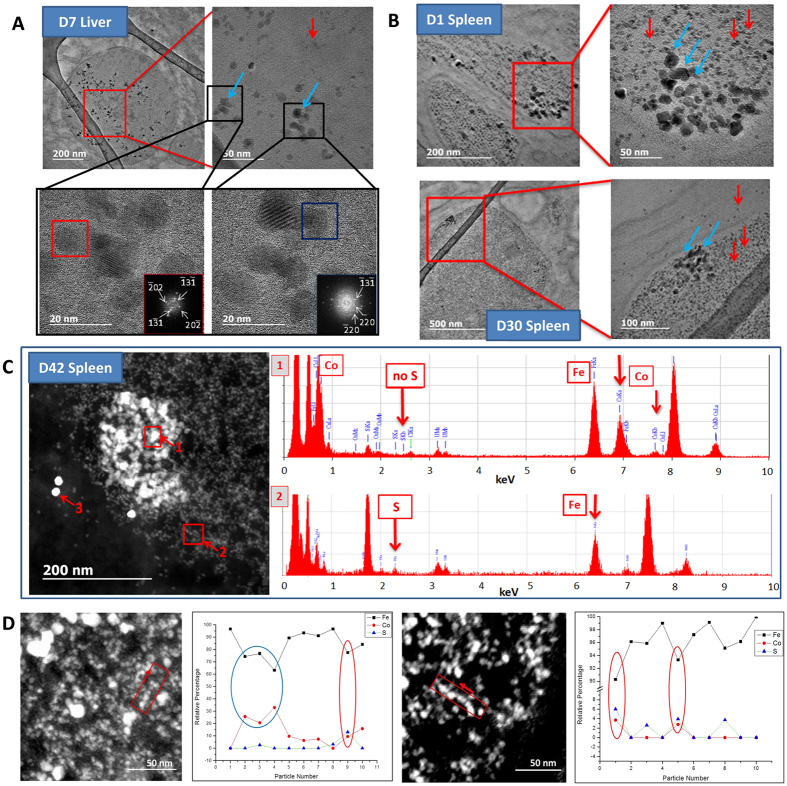
TEM tracking and EDX nano analyses of CoIONPs and ferritins in liver and spleen at different time-points after intravenous injection of CoIONPs. (**A**) CoIONPs are identified in the lysosomes of liver macrophages at day 7, by their characteristic size distribution (8.7 ± 2.9 nm) and inverse spinel structure of cobalt ferrite, determined from the FFT of the HRTEM pictures (red and blue squares). (**B**) CoIONPs (blue arrows) are seen in the neighborhood of numerous HoloF proteins (red arrows) in spleen lysosomes at day 1 (top images) and at day 30 (bottom images). (**C**) STEM-HAADF micrograph of intralysosomal CoIONPs in spleen at day 42 post injection. The 10 nm monodisperse bright particles (labelled 3) are antiferritin immunogold particles. The less bright and polydisperse particles (square “1”) are the native CoIONPs with a Fe:Co ratio of 2 to 3 and absence of sulfur, as determined by EDX analysis. The small quasi-monodisperse particles correspond to HoloF, characterized by the colocalization of iron and sulfur (Fe:S ratio of about 20). (**D**) Single particle EDX analyses on spleen samples at day 42 post-injection. The graphs on the right of the STEM-HAADF picture show the relative percentage of Fe, Co and S determined from the point by point EDX analysis of the red quadrant scanned in the arrow direction. Blue oval depicts a native CoIONP particle, while red ovals indicate colocalization of cobalt, iron and sulfur, indicating cobalt uptake by ferritins.

Citrate-coated CoIONPs of 8.7 ± 2.9 nm diameter synthesized by coprecipitation method (Figure S1) were injected intravenously in mice at the dose recommended for clinical MRI when using IONPs (50 μmol/kg iron). Structural follow up of CoIONPs was performed by TEM, High Resolution TEM (HRTEM) and scanning TEM using a high angle annular dark field detector (STEM-HAADF) in liver and spleen samples (Figs 1 and 2) up to 8 months post-injection. In parallel, the Co content was measured by inductively coupled plasma-mass spectrometry (ICP-MS) in these organs (Figure S2). Histological examinations of liver, spleen and kidney of treated animals at day 1, day 7 and day 34 after injection of CoIONPs do not show any microstructural alterations in comparison to control, untreated mice (Figures S3–S6). Co content in spleens and livers decreases over time (more rapidly in the liver) but a substantial fraction of the cobalt content at D1 (about 30%) still persists in spleen after 4 months. Similar results were found previously for IONPs with different shapes, sizes and surface coating^13^,^34^,^35^. Both the excretion of intact nanoparticles or the transfer of Co to different organs or metabolic pools may account for the decrease in the Co contents in liver and spleen with time. Consistent with elemental analysis, TEM observations at the nanoscale show intact CoIONPs in the lysosomes of splenic and hepatic macrophages from day 1 to 8 months post-injection, although the intact particles tend to rarify over time (Fig. 1 and Figure S7). Iron-filled ferritins are distinguished from native CoIONPs first by their characteristic spherical shape and rather monodisperse size, up to eight nanometers, and second by the use of colloidal gold immunological labeling with anti-ferritin antibodies (Fig. 1). Numerous ferritin proteins are observed in the neighborhood of CoIONPs lying within lysosomes (Fig. 1), with similar patterns to what was previously observed after injection of IONPs^3^,^4^,^5^. Ferritins are either found as large organized clusters or are individually disseminated within lysosomes or within the cytoplasm. Structural and elemental analyses of the exogenous intact or degraded CoIONPs and endogenous ferritin proteins were achieved by HRTEM, STEM-HAADF and energy dispersive X-ray (EDX) nano-analyses performed on single particles at different time-points after injection. Apart from their distinct sizes, structures and contrast in comparison to CoIONPs, ferritin proteins are identified by EDX through their sulfur content. In contrast, injected CoIONPs or their remnants are characterized by colocalization of cobalt, iron, and an absence of sulfur (Fig. 2C). Importantly EDX also reveals the nature of the metals incorporated inside the ferritin protein shell. While most of ferritins contain only iron (collocating with sulfur), some of them reveal the presence of cobalt collocating with iron and sulfur (Fig. 2D). Although the quantities of cobalt in ferritins are much smaller than the iron ones, these observations in spleen samples, 42 days after injection, support the hypothesis that ferritins could incorporate both iron and cobalt ions, transferred from CoIONPs ferritins within lysosomes, providing the first observation of metal ions transfer from exogenous nanoparticles to endogenous ferritin proteins *in vivo*.

**Figure 3:**
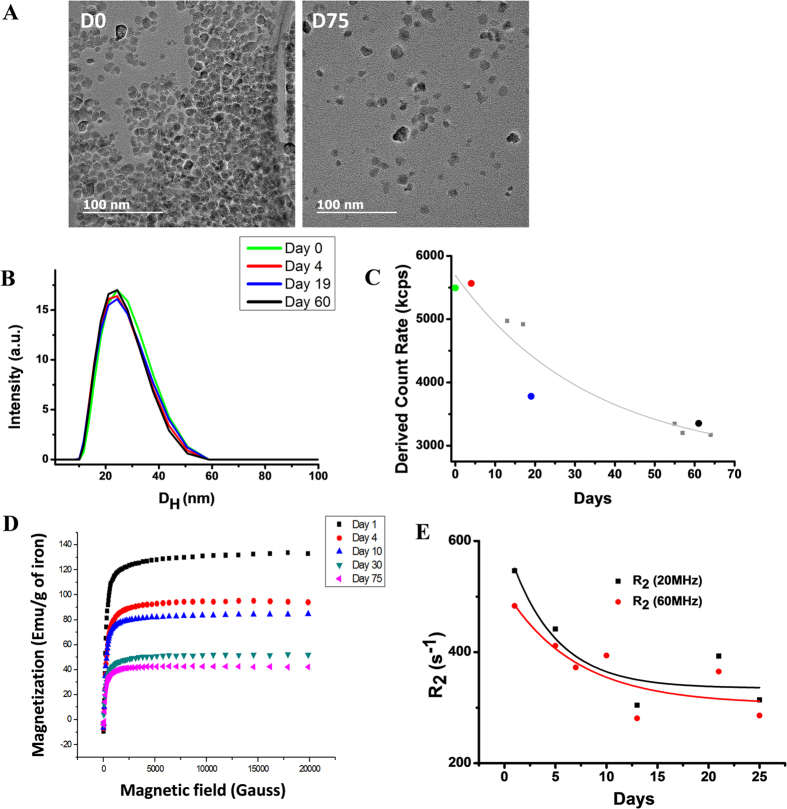
Evolution of citrate-coated CoIONPs in acidic citrate medium. (**A**) Representative TEM micrographs at two time-points. (**B**) Time evolution of the distribution of hydrodynamic sizes and (**C**) derived count rate in DLS. (**D**) Time evolution of the field-dependent magnetization curve at 300 K and (**E**) Time evolution of the transverse NMR relaxation rate R_2_ at proton Larmor frequencies of 20 MHz and 60 MHz.

The results suggested by this *in vivo* study motivated additional *in vitro* investigations to probe the degradability of CoIONPs in a simplified model of lysosomal environment and the ability of ferritin to integrate cobalt and iron ions in the acidic pH, which is present within lysosomes, but was formerly thought to hinder the binding of metals other than iron to ferritin proteins^31^. IONPs were previously shown to degrade in a minimal medium mimicking the acidic lysosome environment and consisting of sodium citrate (10 mM) at pH = 4.7^5^,^42^,^43^,^44^,^45^. Here we also observe the degradation of CoIONPs in this acidic citrate medium (Fig. 3). The loss of magnetic properties of CoIONPs over time is attested by the decrease of the saturation magnetization in magnetization curves (Fig. 3D) together with a decrease of the transverse nuclear magnetic resonance (NMR) relaxation rate R_2_ at proton Larmor frequencies of 20 MHz and 60 MHz, which probes the efficiency of CoIONPs as MRI contrast agents (Fig. 3E). The hydrodynamic size distribution of CoIONPs assessed by diffusion light scattering (DLS) remains almost constant over time, but the time-averaged light scattered intensity (Fig. 3B,C) decreased, which is consistent with a diminution of the number of particles. HRTEM and EDX elemental analysis on single particles indicate that the spinel structure of the remaining particles is maintained during the degradation and the fraction of Co per Fe in particles (Co/Fe = 40/60) remains unchanged up to day 75.

**Figure 4:**
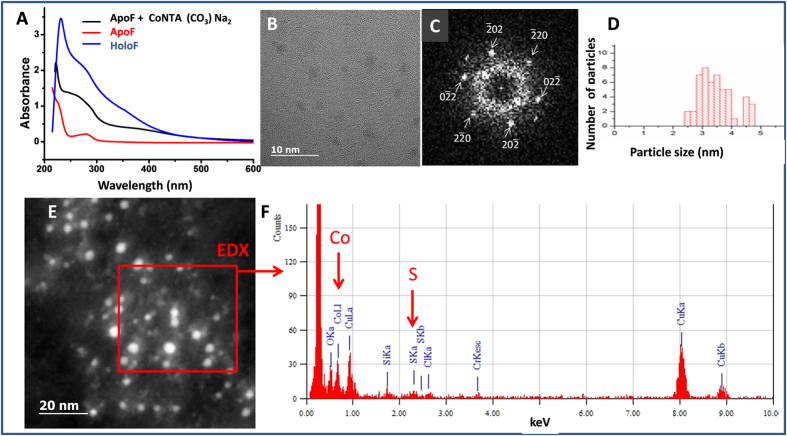
Signature of apoferritin filling cobalt from cobalt salt CoNTA (CO_3_) Na_2_. (**A**) UV-Vis spectrum of ApoF incubated with CoNTA (CO_3_) Na_2_ for 22 hours in acidic medium in comparison to ApoF alone and HoloF. Note the growth of the absorbance shoulder at 280 nm indicating the filling of the protein cage. (**B**) HRTEM of ApoF incubated with CoNTA showing crystalline structures with diffraction pattern (**C**) characteristic of the cobalt oxide (CoO) along [111] zone axis. (**D**) Distribution of crystal sizes (mean diameter 3.5 ± 0.6 nm) suggesting a partial filling of the protein cage, in line with UV-Vis spectrum. (**E**) STEM-HAADF images of ferritin nanocrystals and (**F**) EDX analysis of the red area, indicated characteristic lines of cobalt and sulfur.

We next investigated whether ApoF protein core is capable of accommodating Co ions coming from cobalt salt in lysosome-mimicking acidic citrate medium. Cobalt salt CoNTA(CO_3_)Na_2_ was incubated with ApoF for one day in acidic citrate medium and subsequently dialyzed. The UV-visible spectrum of the mixture was monitored at different time-points and compared to the UV-vis signatures of ApoF and iron-filled HoloF. The intensity increase of the band at 220 nm and the evolution of the band at 280 nm indicates a metal-protein bond (Fig. 4)^37^,^46^. The UV-vis spectra of Co salt/ApoF mixture show a clear evolution toward the absorbance signal of commercial HoloF suggesting a filling of ApoF with cobalt. This result is confirmed by HRTEM, which shows the presence of crystalline NPs with a diameter of 3.5 ± 0.6 nm (60% of the commercial HoloF size) and CoO structure. The EDX analysis in the STEM-HAADF mode indicates the colocalization of sulfur and cobalt and confirms the storage of cobalt in ApoF (Fig. 4). The formation of cobalt oxide Co_3_O_4_ was previously reported, but the result was obtained in a different pH range^37^. Overall, our results suggest a partial filling of ApoF cavity with cobalt even at the low pH value^47^.

**Figure 5:**
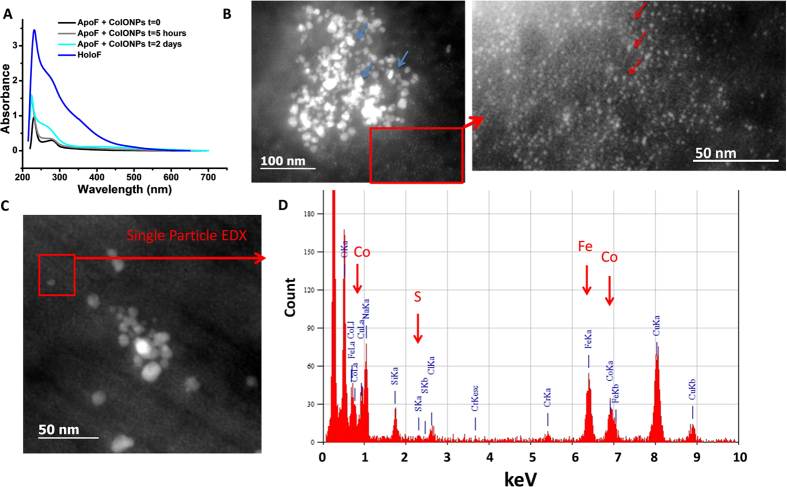
Metal transfer from NPs to apoferritin proteins. (**A**) UV-vis spectra of ApoF incubated with CoIONPs in acidic medium for 5 hours and 2 days, in comparison to ApoF and HoloF. The growth of absorbance shoulder at 280 nm indicates metal filling of the protein. (**B**) STEM-HAADF images of CoIONPs (blue arrows) incubated with ApoF for 2 months. Metal-filled proteins appear as small particles (red arrows). (**C**,**D**) Single particle EDX analysis of the red-contoured area in C confirms occupancy of iron, cobalt and sulfur in single ApoF with relative atomic percentages of 64.7%, 28.2% and 7.13% respectively.

The presence and the composition of ferritins nearby CoIONPs in organ slices suggest a transfer of iron and cobalt ions relocating from NPs to ferritins. We thus analyzed the evolution of ApoF interacting with CoIONPs in acidic citrate medium. During the two days of incubation at 37 °C, the absorbance signal of ApoF evolve to that of the commercial HoloF, showing an incremental filling of ApoF with metals transferred from NPs (Fig. 5). STEM-HAADF and EDX analysis confirm the presence of two types of structures: the native cobalt ferrite NPs (8.7 ± 2.9 nm) coexisting with very small NPs (3.3 ± 0.4 nm) which exhibit EDX peaks for iron, cobalt and sulfur at 6.45 KeV, 6.95 KeV and 2.4 KeV, respectively, with relative atomic percentages of 64.7%, 28.2% and 7.13% and with an error of ±1%. This result clearly confirms the ability of ApoF to sequester simultaneously iron and cobalt ions coming from the degradation of CoIONPs within a minimal lysosome-like environment.

## Conclusion

In conclusion, elemental nanoscale investigations of excised organs highlighted the possible implication of endogeneous ferritin proteins in the recycling of iron and cobalt originating from intralysosomal degradation of cobalt ferrite NPs. In previous studies, high resolution observations of excised organs provided information on the ultrastructure of lysosomes in macrophages located within different tissues (spleen, liver^3^,^5^,^15^, inflammatory sites such as adipose tissue^48^ or atherosclerotic plaques^14^) and pointed out the ubiquitous presence of HoloF nearby or within IONP-rich zones in organs harvested from treated animals. In addition, the local lysosomal degradation of IONPs has been empirically demonstrated^4^ and the transfer of ^59^Fe from IONPs to the hemoglobin of rats^6^ and mice^7^ was established. On the other hand, ferritins are able to antagonize lysosomal iron overload and oxidative stress by being relocalized in lysosomes or synthesized *de novo*^29^,^49^,^50^. However neither the transfer of metal ions from nanoparticles to ferritin was formally demonstrated, nor the transfer within environments exhibiting acidic pH going below 5.5 has been reported. Yet, due to biological intralysosomal sequestration and processing of nanoparticles, the transfer to ferritins in acidic media has a particular physiological relevance. In the present study EDX nanoanalysis provided evidence for the first time, that intracellular ferritin cages localized nearby degrading CoIONPs could act as storage proteins for both iron and cobalt ions released by injected NPs. The investigations performed *in vitro* in the minimal model of the acidic lysosome environment served to undoubtedly demonstrate that this metal transfer is empirically possible at pH 4.7 with Co coming from metal salts, but also from cobalt ferrite NPs degraded in the acidic citrate medium. *Ex vivo* evidence for iron and cobalt remediation after *in vivo* administration of cobalt ferrite nanoparticles suggests that ferritins could represent an archetypal mechanism of metabolization of metal-containing NPs.

## Experimental Section

### Nanoparticles

CoIONPs are synthetized by co-precipitation of Co(II) and Fe(III) hydroxide^51^. CoIONPs (CoFe_2_O_4_) have the inverse spinel structure like magnetite (Fe_3_O_4_) where Fe^2+^ ions are substituted by Co^2+^ ions. CoIONPs are coated by citrate ligands. The suspensions were stable in water in a pH range of 3–8 owing to electrostatic stabilization (negatively charged citrate-coating). The size distributions of the NPs were determined by TEM. The hydrodynamic diameter was determined by DLS in water and was in the range of 20 to 40 nm. Detailed NPs specifications are reported in supporting information Figure S1.

### *In vivo* study

Animal experiments were approved by the ethical comity of University Paris Descartes and performed in accordance with institutional ethical rules and guidelines for animal use and care of the Paris Cardiovascular Research Center animal facility. The animals were allowed to acclimate to this facility for at least 1 week before being used in the experiments and were fed a standard diet *ad libitum* throughout the experiments. Pathogen-free female 8 week old C57/Bl6 mice (mean weight 20.5 ± 1 g) (Janvier, France)) were injected in the retro-orbital vein with CoIONPs suspended in 100 μL of physiological saline medium at a dose of 50 μmol iron/kg (injected dose of 56 μg iron). One mouse was sacrificed at each time point after CoIONPs injection (at days D1, D7, D30, D42 and D112, respectively). Livers and spleens were excised and prepared for TEM and ICP-MS experiments.

### Inductively coupled plasma-mass spectrometry (ICP-MS)

Cobalt content in suspension and organs was quantified by ICP-MS. After excision, organ samples were conserved at −80 °C until preparation for ICP-MS measurements. The organs were mineralized in closed vials in presence of 4 ml nitric acid at 70 °C for 2 h on heating blocs (DigiPREP Jr SCP Science, Canada) and then heated in open vials at 80 °C for 10 additional hours. The volume of each sample was adjusted to 20 or 4 ml with 3% nitric acid in distilled water and analyzed for Co content by ICP-MS (Thermo Electron, France).

### Transmission electron microscopy (TEM)

Organs were cut into 1 mm^3^ pieces after excision and fixed with 2% glutaraldehyde in 0.1 M sodium cacodylate buffer, post-fixed with 1% osmium tetroxide containing 1.5% potassium cyanoferrate, gradually dehydrated in ethanol, and embedded in Epon. Thin sections (70 nm) of selected zones were observed with Zeiss EM902 electron microscope operated at 80 kVe (MIMA2 MET –GABI, INRA, Jouy-en-Josas, France). Ultrathin sections (30 nm) were prepared for high-resolution imaging and EDX analysis.

High resolution TEM imaging and EDX spectroscopy were performed on a JEOL ARM 200 F microscope^52^, equipped with a CEOS aberration corrector, a cold field emission gun and a JEOL EDX diode, operated at 200 kV or 80 kV (biological samples).

### Gold immunolabeling on TEM sections

Tissues were fixed with a mixture of 4% paraformaldehyde in 0.1 M phosphate buffer, pH 7.2. The 70 nm sections were quenched with glycine 50 mM in 0.1 M phosphate buffer pH 7.2, blocked with buffer containing 10% Goat normal serum (GNS), 1% bovine serum albumin (BSA), 0.1% BSA-c^TM^ (prepared by acetylation of BSA (Aurion, Nederland)). Antiferritin antibodies were added to a 1/100 w/w dilution in buffer containing 1% GNS, 1% BSA, 0.1% BSA-c^TM^ and incubated for 2 h; the grids were rinsed twice, then the goat anti-rabbit IgG coupled to 10 nm colloidal gold particles (British Biocell International – TEBU, France) was used at a 1/50 dilution for an 1 hour incubation. The grids were rinsed again, and stained with 2% uranyl acetate. Grids were examined with a Hitachi HT7700 electron microscope operated at 80 kV (Elexience – France), and images were acquired with a charge-coupled device camera (AMT).

### Lysosome-like buffer

The medium used to mimic the acidic environment of the lysosomes, as described by Arbab *et al*.^43^, consisted of 20 mM citric acid at pH 4.7. The acidic citrate buffer was prepared by mixing 10 mM of citric acid (C_6_H_8_0_7,_ Fluka, France, >99, 5%) and 10 mM of sodium citrate tribasic (C_6_H_5_Na_3_O_7_. 2H_2_O, Fluka, >99%) in 250 mL of purified water.

### Metal transfer to ApoF

#### Filling of ApoF with metal complex salts

A 0.05 mM solution of ApoF (apoferritin from equine spleen, Sigma, France) was incubated at 37 °C for one day with an excess of the mixed sodium cobalt(III) nitrilotriacetate carbonate complex Na_2_CoNTA(CO_3_) in the acidic citrate buffer and the mixture was dialyzed (Dialysis membrane 50 kD, Spectrum Labs, France) to remove the excess of cobalt complex salt.

#### Filling ApoF with NPs

The NPs were incubated with ApoF in the acidic citrate buffer for different times, the final concentrations being 0.12 mM of NPs and 0.5 μM of ApoF.

### UV-visible Spectroscopy

Absorption measurements were performed at (37 ± 0.5)°C on a Cary 4000 UV/Vis/NIR spectrophotometer equipped with a thermostated cell-carrier. The signal of non-degraded NPs was subtracted to remove the diffusion signal related to NPs.

### *In vitro* NP degradation procedure

NPs were incubated at 37 °C in the dark with and without ApoF in the acidic citrate buffer, at iron + cobalt concentration of 10 mM. ApoF was added at a concentration of 2.2 μm (corresponding to 4500 iron atoms available for one ApoF).

### Nuclear Magnetic Resonance Dispersion (NMRD)

The frequency dependence of the longitudinal ^1^H relaxation rate, R_1_ = 1/T_1_, was recorded in the suspension over the frequency range of 0.015 to 40 MHz using a Spinmaster FFC-2000 fast-field cycling NMR relaxometer (Stelar SRL, Italy). The temperature of the samples was maintained at 37 °C using a thermostated airflow system. All of the ^1^H magnetization recovery curves were mono-exponential within experimental error. R_2_ were measured using the CPMG pulse sequence with an echo time of 1 ms on a 20 MHz and on a 60 MHz Bruker Minispec. All measurements were performed in samples with iron + cobalt concentration of 1 mM, prepared by aqueous extemporaneous 10-times dilutions of the suspensions in acidic citrate medium taken at different incubation times.

### Dynamic Light Scattering (DLS)

DLS measurement were carried out at 25 °C on a Zeta Sizer Nano ZS (Malvern Instruments, France) equipped with a 5.0 mW He-Ne laser operating at 632.8 nm and an Avalanche photodiode detector. The time-averaged intensity of scattered light at 173 degrees (derived count rate) was used to quantify the number of scattering NPs in course of their degradation. We also verified that for the native non-degraded NPs, the time-averaged intensity of scattered light was proportional to the iron + cobalt concentration. The dispersions were never filtered before measurements.

### Magnetic measurements

Magnetization measurements were carried out on a vibrating sample magnetometer (PPMS, Quantum Design, Inc., CA, USA) on suspensions of CoIONPs at 10 mM iron + cobalt concentration in acidic citrate medium. The data were corrected from the diamagnetic contribution of the acidic citrate buffer and of the sample holder. The field-dependent magnetization curves were measured at 310 K in the range between 0 and 3 × 10^4^ Gauss.

## Additional Information

**How to cite this article**: Volatron, J. *et al*. Physiological Remediation of Cobalt Ferrite Nanoparticles by Ferritin. *Sci. Rep.* **7**, 40075; doi: 10.1038/srep40075 (2017).

**Publisher's note:** Springer Nature remains neutral with regard to jurisdictional claims in published maps and institutional affiliations.

## Supplementary Material

Supplementary Information
