# Supplementary material for: Physiological Remediation of Cobalt Ferrite Nanoparticles by Ferritin
Source: Sci Rep. 2017 Jan 9;7:40075. doi: 10.1038/srep40075 (PMC5220348; doi:10.1038/srep40075)
Supplement: Supplementary Information [file srep40075-s1.pdf]

Supplementary materials

Physiological Remediation of Cobalt Ferrite Nanoparticles by Ferritin

Jeanne Volatron,[1] Jelena Kolosnjaj-Tabi,[1,2] Yasir Javed,[3] Quoc Lam Vuong,[4] Yves Gossuin,[4] Sophie Neveu,[5] Nathalie Luciani,[1] Miryana Hémadi,[6] Florent Carn,[1] Damien Alloyeau,[3] Florence Gazeau[1]

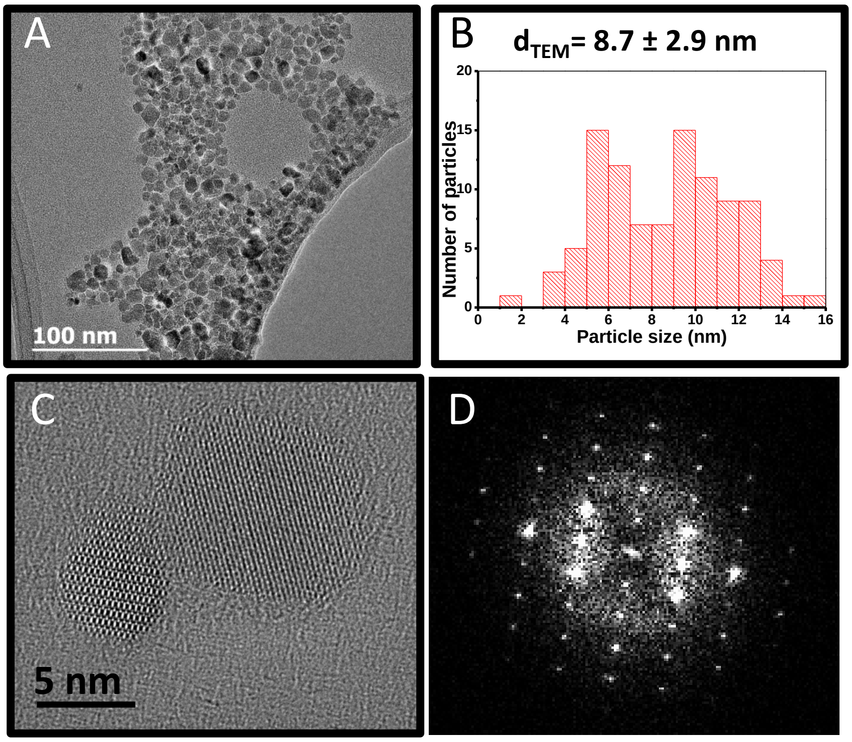

Figure S1: **Characteristics of CoIONPs.** A) TEM images of citrate-coated CoIONPs and B) corresponding TEM size distribution with a mean diameter of (8.7 ± 2.9 nm). C) High resolution images of CoIONPs and D) FFT of selected particles show monocrystalline inverse spinel structure oriented along the [112] zone axes.

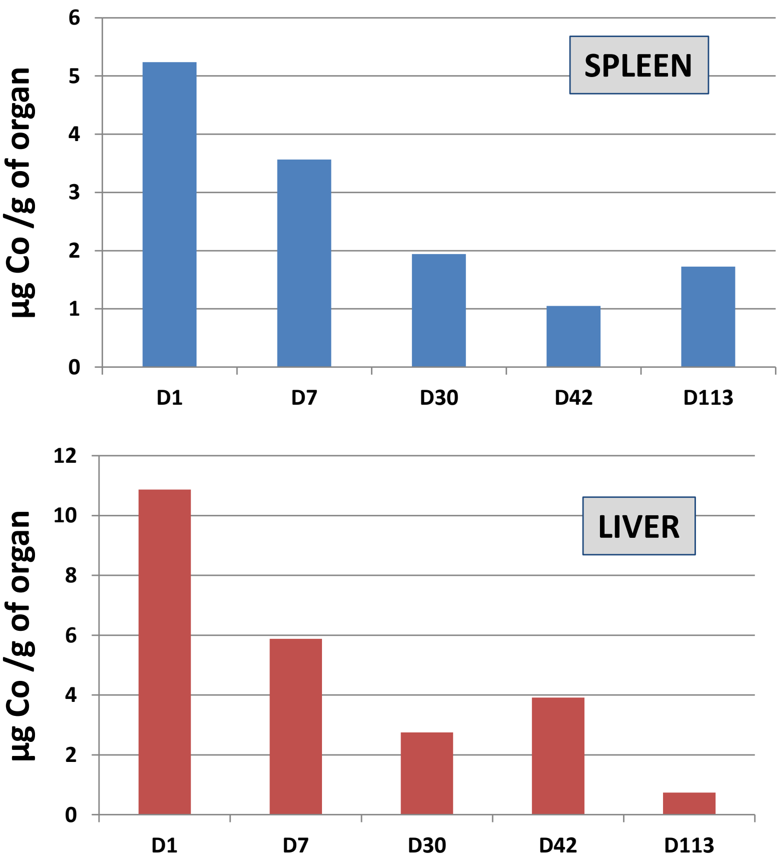

Figure S2: ICP-MS quantification of Co content in spleen and liver of mice injected with CoIONPs (50 µmol iron/kg body weight) at different time-points after injection indicated in days. The amount of Co is expressed as µg Co per g of fresh organ. Note the diminution over time of the cobalt content, but also the persistence in spleen of one third of the Co content at day 1, almost 4 months after injection.

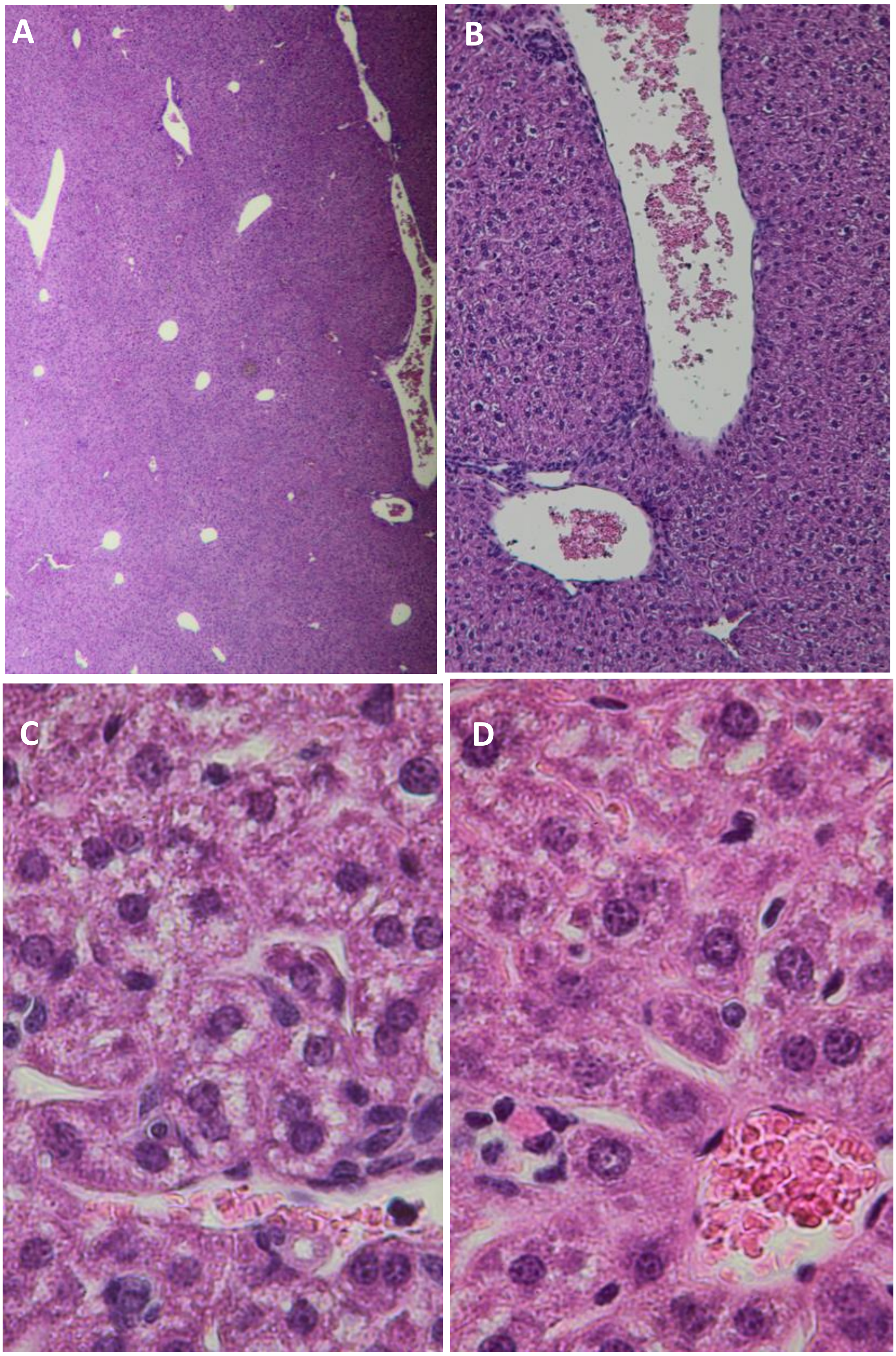

Figure S3: Histological staining (hematoxylin and eosin) of mouse liver at day 1 post-injection of nanoparticles (A, B, C) and in control non-injected mouse (D). Magnification are 2.5 (A), 10 (B) and 63 (C&D).

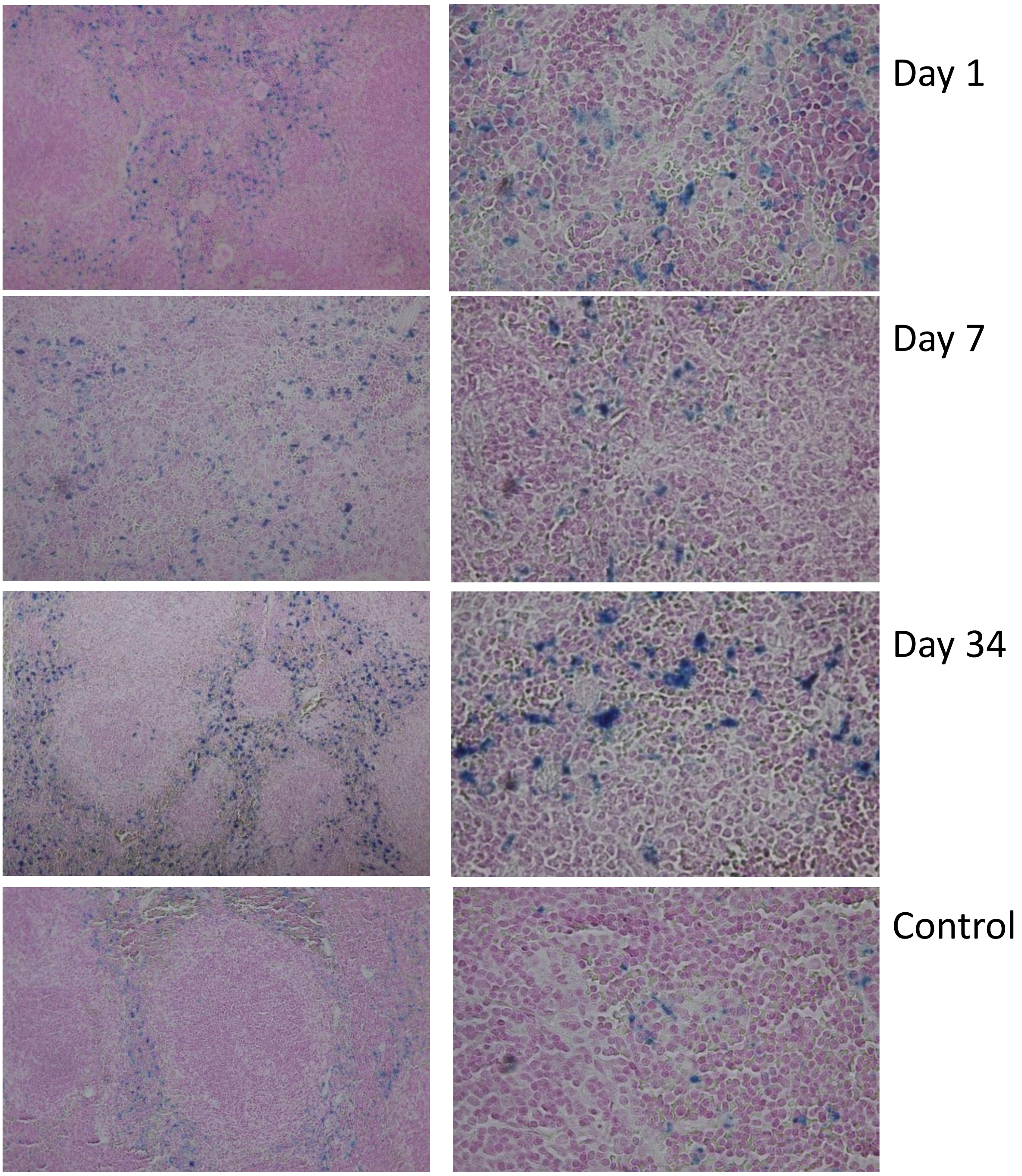

Figure S4: Histological staining (Pearls staining highlighting iron in blue) of mouse spleen at day 1, day 7 and day 34 post-injection of nanoparticles and in control non-injected mouse. Magnifications are x10 (left) and x20 (right).

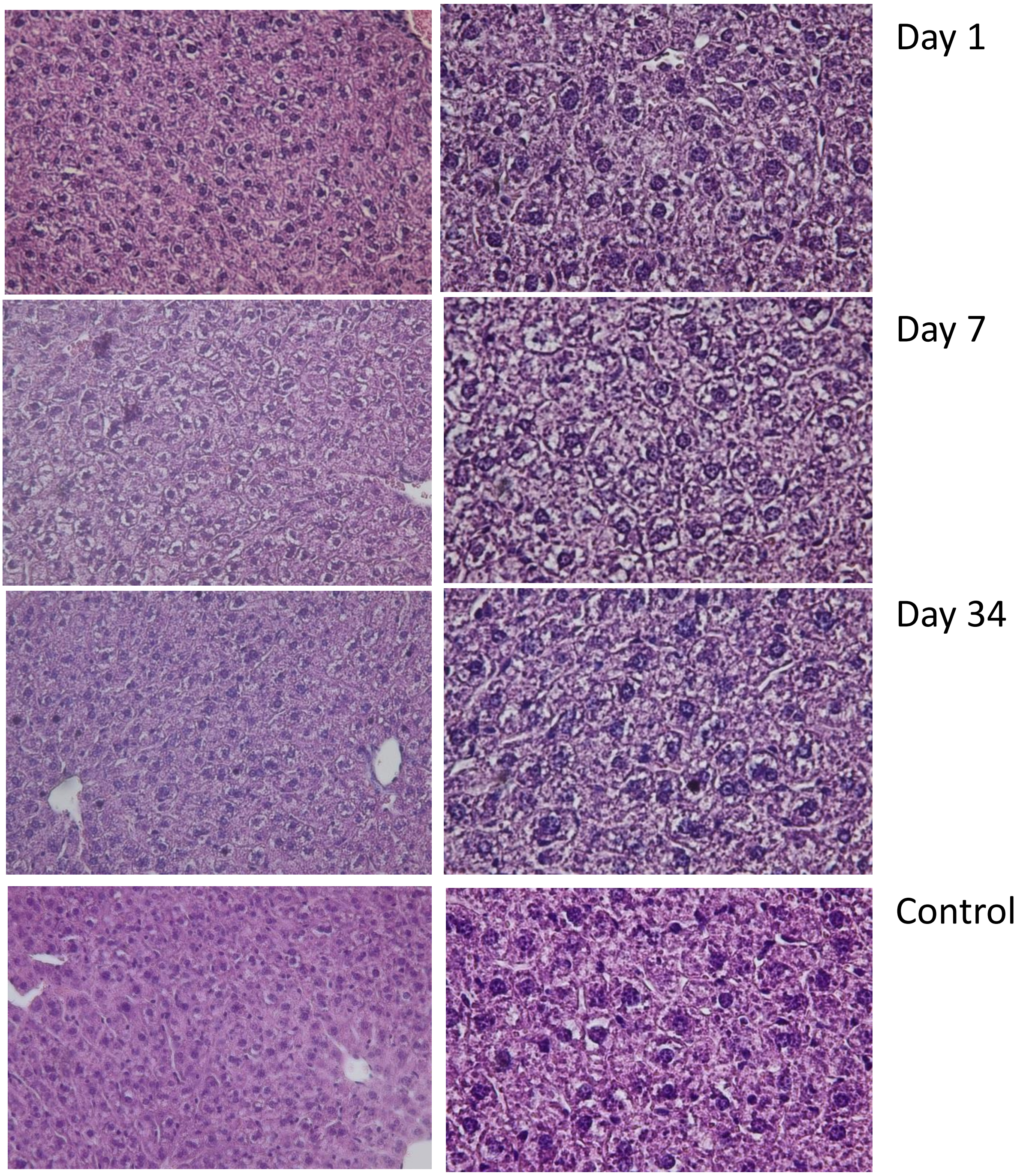

Figure S5: Histological staining (hematoxylin and eosin) of mouse liver at day 1, day 7 and day 34 post-injection of nanoparticles and in control non-injected mouse. Magnification are x20 (left) and x40 (right).

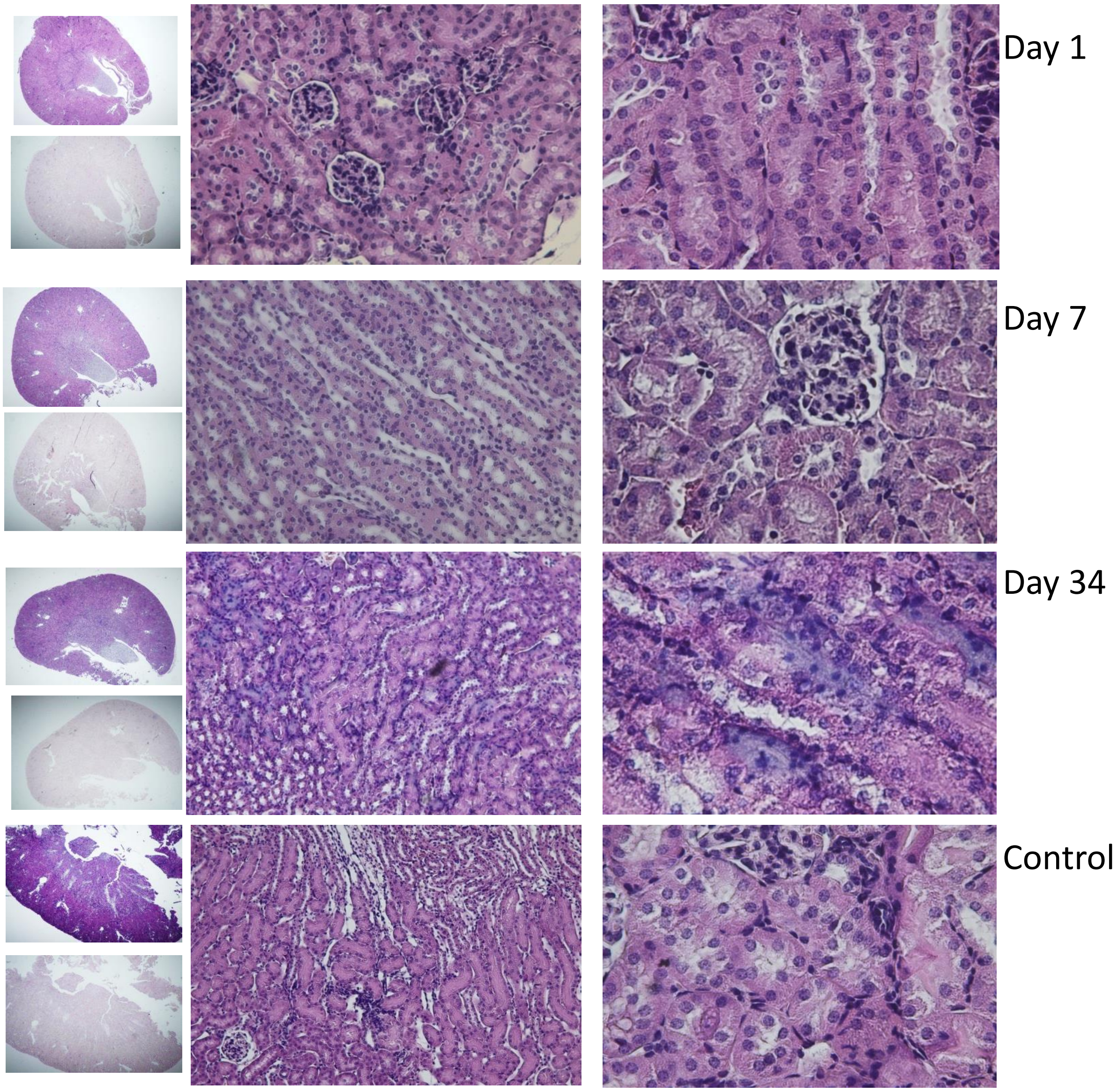

Figure S6: Histological staining (hematoxylin and eosin) of mouse kidney at day 1, day 7 and day 34 post-injection of nanoparticles and in control non-injected mouse. Magnification are x1.6 (left), x20 (middle) and x40 (right).

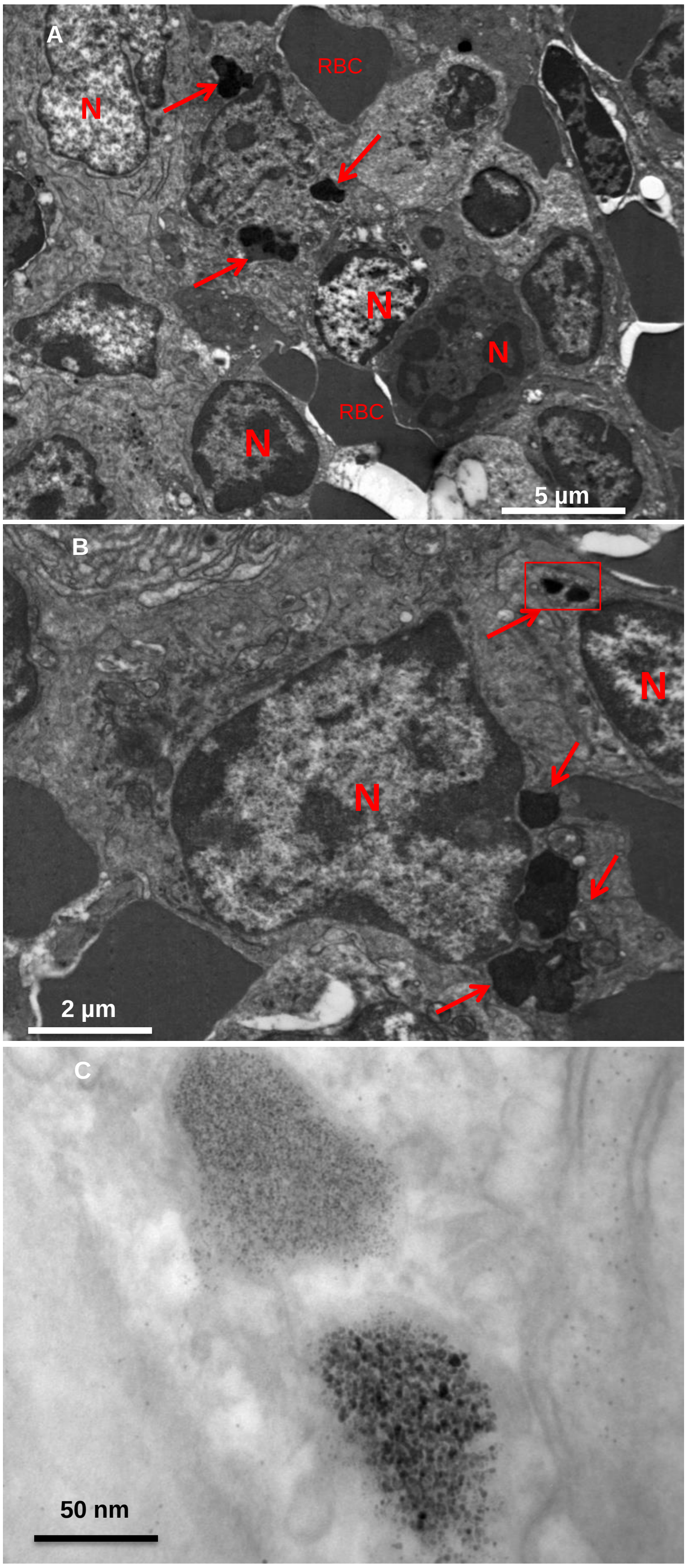

Figure S7: Electron microscopy general and magnified view of the spleen at D34 post injection. N=nucleus, arrow nanoparticle-rich zone, RBC = red blood cell. The red sqare in B is magnified in C and it reveals two families of nanoparticles that exhibit different sizes and electron density, probably pertaining to ferritins (upper) and cobalt ferrite nanoparticle remnants (lower group).
